# Enhanced Prefrontal Regional Homogeneity and Its Correlations With Cognitive Dysfunction/Psychopathology in Patients With First-Diagnosed and Drug-Naive Schizophrenia

**DOI:** 10.3389/fpsyt.2020.580570

**Published:** 2020-10-23

**Authors:** Shuzhan Gao, Yidan Ming, Jiayin Wang, Yuan Gu, Sulin Ni, Shuiping Lu, Rongrong Zhang, Jing Sun, Ning Zhang, Xijia Xu

**Affiliations:** ^1^Department of Psychiatry, Affiliated Nanjing Brain Hospital, Nanjing Medical University, Nanjing, China; ^2^Department of Psychiatry, Nanjing Brain Hospital, Medical School, Nanjing University, Nanjing, China

**Keywords:** schizophrenia, regional homogeneity (ReHo), cognitive dysfunction, support vector machine analysis, prefrontal

## Abstract

**Background:** Schizophrenia, regarded as a neurodevelopmental disorder, is characterized by positive symptoms, negative symptoms, and cognitive dysfunction. Investigating the spontaneous brain activity in patients with schizophrenia can help us understand the underlying pathophysiologic mechanism of schizophrenia. However, results concerning abnormal neural activities and their correlations with cognitive dysfunction/psychopathology of patients with schizophrenia were inconsistent.

**Methods:** We recruited 57 first-diagnosed and drug-naive patients with schizophrenia and 50 matched healthy controls underwent magnetic resonance imaging. The Positive and Negative Syndrome Scale (PANSS) and the MATRICS Consensus Cognitive Battery were used to assess the psychopathology/cognitive dysfunction. Regional homogeneity (ReHo) was used to explore neural activities. Correlation analyses were calculated between abnormal ReHo values and PANSS scores/standardized cognitive scores. Lastly, support vector machine analyses were conducted to evaluate the accuracy of abnormal ReHo values in distinguishing patients with schizophrenia from healthy controls.

**Results:** Patients with schizophrenia showed cognitive dysfunction, and increased ReHo values in the right gyrus rectus, right inferior frontal gyrus/insula and left inferior frontal gyrus/insula compared with those of healthy controls. The ReHo values in the right inferior frontal gyrus/insula were positively correlated with negative symptom scores and negatively correlated with Hopkins verbal learning test-revised/verbal learning. Our results showed that the combination of increased ReHo values in the left inferior frontal gyrus/insula and right gyrus rectus had 78.5% (84/107) accuracy, 85.96% (49/57) sensitivity, and 70.00% specificity, which were higher than other combinations.

**Conclusions:** Hyperactivities were primarily located in the prefrontal regions, and increased ReHo values in the right inferior frontal gyrus/insula might reflect the severity of negative symptoms and verbal learning abilities. The combined increases of ReHo values in these regions might be an underlying biomarker in differentiating patients with schizophrenia from healthy controls.

## Introduction

Schizophrenia, a psychiatric syndrome affecting 0.6% of the population in China, is characterized by positive symptoms (hallucination, delusions, and disorganization symptoms), negative symptoms (hypobulia, anhedonia, affective blunting, social withdrawal, and alogia), and cognitive dysfunction (processing speed, attention/vigilance, working memory, etc.) (1, 2), however, its etiology is still unclear, and diagnosis primarily relies on psychopathology. To date, schizophrenia is regarded as a neurodevelopmental disorder, and its symptoms occur spontaneously. Therefore, investigating spontaneous brain activities in patients with schizophrenia can help us understand the potential pathophysiologic mechanism of schizophrenia (3).

Resting-state functional magnetic resonance imaging (MRI), as a non-invasive examination, has been applied to explore the neural activity by recording signals dependent on blood oxygenation levels. Several studies have focused on functional connectivity (FC) analyses and revealed that subjects with a high risk of schizophrenia and first-episode schizophrenia showed a shared aberrant FC in the prefrontal cortex (PFC); those findings have indicated that abnormalities occur before disease onset, and abnormal neural activities in this region may be a trait alteration of schizophrenia (4, 5). In addition, a review has demonstrated that abnormal connections are found in patients with schizophrenia between the PFC and other regions, such as the basal ganglia, temporal regions, parietal regions, hippocampus, and default mode network; dysconnectivity between regions is associated with cognitive dysfunction and psychopathology (6). However, studies on FC have investigated “distinct” brain areas and hardly reflected “local” synchronization (3). In contrast to traditional FC, local FC based on neurodevelopment can be used to measure the functional interactions or synchronization of neighboring voxels. Local FC also affects remote FC and whole brain dynamics, highlighting the importance of exploring local FC (7–10).

Regional homogeneity (ReHo) can be used to measure the similarity or synchronism of the time series within neighboring voxels and reflect the coordination of regional neural activities. Increased and decreased ReHo values represent abnormal neural activities (11). According to previous studies on subjects with a high risk of schizophrenia (12, 13), first-episode adolescent-onset drug-naïve schizophrenia (14, 15), first-episode drug-naïve schizophrenia (16), chronic schizophrenia (17), and treatment-resistant schizophrenia (18), abnormal ReHo may be a good biomarker to distinguish patients with schizophrenia from healthy controls with increased or decreased ReHo values in different regions. However, previous results were inconsistent. Moreover, abnormal neural activities are associated with clinical symptoms and cognitive dysfunction, and ReHo may be used to evaluate the severity of clinical symptoms and cognitive dysfunction. With regard to clinical symptoms, abnormal ReHo values in several brain regions of patients with schizophrenia are positively/negatively/not associated with the Positive and Negative Syndrome Scale (PANSS) total score, positive factor, disorganized/concrete factor, excited factor and depressed factor (14–16, 19). In term of cognitive dysfunction, a study on subjects with a genetically high risk of schizophrenia has demonstrated that delayed recall is negatively associated with decreased ReHo values in the right superior frontal gyrus. Uncoupled relationships in patients with schizophrenia between abnormal ReHo values in several regions and attention impairments are found (20). Fluency scores and stroop color-word test scores are related to abnormal ReHo values (14, 15). These findings have suggested that investigating the spontaneous brain activities and their relationships with cognitive dysfunction/psychopathology in patients with schizophrenia can help us understand the potential pathophysiologic mechanism of schizophrenia.

In our study, we hypothesized that abnormal neural activities could be found regionally in the PFC, and these abnormities could reflect the severity of cognitive dysfunction and psychopathology. We might offer insights into the pathophysiologic mechanism of schizophrenia and develop a biomarker to distinguish patients with schizophrenia from healthy controls. First-diagnosed and drug-naïve patients with schizophrenia were recruited to explore the ReHo values in the whole brain and its correlations with cognitive dysfunction/psychopathology and to eliminate the interference of an antipsychotic drug. Computer-based analysis was conducted to re-evaluate the diagnosis of schizophrenia by using ReHo values. Support vector machine (SVM) analyses, an optimized classification method, is applied to classify and diagnose the disease (21). Therefore, using SVM to calculate the accuracy of abnormal ReHo values could help us distinguish patients with schizophrenia from healthy controls.

## Methods

### Subjects

For patients with schizophrenia, 60 right-handed inpatients with schizophrenia were recruited from Affiliated Nanjing Brain Hospital, Nanjing Medical University, from April 2018 to December 2019. Schizophrenia was co-diagnosed by two chief psychiatrists in accordance with the International Classification of Diseases, 10th Revision (ICD-10: F20) using the MINI-International Neuropsychiatric Interview for ICD-10 diagnoses. The severity of symptoms and cognitive dysfunction were assessed with the PANSS (22) and MATRICS Consensus Cognitive Battery (MCCB) (23, 24), respectively, in the first interview. Moreover, the Annett Hand Preference Questionnaire was utilized to assess right-handedness. Patients were eligible if they met the following inclusion criteria: (1) patients aged at 16–60 years, (2) patients satisfying met the ICD-10 criteria for schizophrenia and PANSS total score of ≥60, and (3) first-time diagnosed and drug-naïve (without any psychiatric treatment). Patients were excluded if they satisfied the following exclusion criteria: (1) patients with organic disorders, psychoactive substances, mood disorder, transient psychotic disorder, and intellectual disability; (2) patients who could not perform a MRI scan, and patients with other brain diseases, such as brain tumors, intra-abscesses, and cerebral infarction; (3) patients who used psychiatric medication ever.

For the healthy controls, 52 right-handed healthy controls were recruited from the community via advertisement. The inclusion criteria were as follow: (1) race, sex, age, and matched patient groups; (2) no mental disorders that met the ICD-10 diagnosis using the MINI-International Neuropsychiatric Interview at present or in the past; and (3) negative family history of mental disorders. The exclusion criteria were as follows: (1) history of severe somatic diseases; (2) cannot perform an MRI or other brain diseases (such as cerebral infarction, brain tumors, demyelinating lesions, and brain abscesses); (3) intellectual disability, IQ <70; and (4) history of alcohol and drug abuse.

This study was approved by the local ethics committee of the Affiliated Nanjing Brain Hospital, Nanjing Medical University (2017-KY017). All the participants and their legal guardians were informed about the procedures with written informed consent.

### Data Acquisition

The demographic and clinical characteristics of the subjects were collected during the first interview. The raw scores of MCCB were standardized with the MCCB software (2014 The Regents of the University of California and SIStat, Version: 3.9.2) mainly in terms of age, sex, and education (25). All the participants were examined with a 3.0T Siemens MRI scanner (Verio, Siemens Medical System) at Affiliated Nanjing Brain Hospital, Nanjing Medical University. Pre-cautions and a birdcage head coil with foam padding were given to all the participants in case of head movement. The scanning parameters were as follows: repetition time (TR) = 2,000 ms; echo time (TE) = 30 ms; FOV = 220 × 220 mm; flip angle = 90°; matrix size = 64 × 64; slice thickness = 4 mm; Gap = 0.6 mm; layers = 33; and time point = 240.

### ReHo Data Processing

Image data were processed using SPM12 (SPM12, Wellcome Department of Imaging Neuro-science, London, UK) and REST (http://resting-fmri.sourceforge.net). Image pre-processing was conducted as follows: (1) except the first 10 time points, (2) slice timing, (3) head motion correction: we excluded the subjects whose maximum displacement of head movement exceeded 2.0 mm in x, y, or z direction or 2° of angular motion and calculated framewise displacement (FD) for each subject and used the mean FD as a covariate in group comparisons, what's more, aggressive head motions (the time points with FD >0.2 mm) were removed to reduce the effect of head motion, (4) spatial normalization, (5) linear trend removing, and bandpass filtering: several sources of spurious variance were then removed from the data using linear regression, including Friston-24 head motion parameters, white matter signal, and cerebrospinal fluid. The data were temporally band-pass filtered (0.01–0.08 Hz) to reduce the effects of low-frequency drift and high-frequency noise.

Regional homogeneity analysis was conducted with the REST software (11). Kendall's coefficient of concordance (KCC) was determined to measure the similarity and consistency of one voxel with those of its nearest neighbors (26 voxels). The KCC map of the whole brain of each participant was calculated. The KCC of each voxel was divided by the average KCC of the whole brain to reduce the individual difference in the whole brain signal. Then, the average KCC of the brain of each participant was obtained, and it corresponded to the average ReHo brain map. Moreover, the averaged ReHo maps were smoothened with a Gaussian kernel of 4 mm full-width at half-maximum to reduce the spatial noise.

### Statistical Analysis

Statistical analysis was conducted using the Statistical Package for Social Science version 24.0 (SPSS 24.0). Age, education, and cognitive scores were evaluated with two-sample *t*-tests between patients with schizophrenia and healthy controls; sex distributions were examined with a Chi-square test. Voxel-based comparisons of the whole-brain ReHo maps with two-sample *t*-tests involving age, sex, years of education, and FD as covariates were performed with the REST software. The Gaussian random field theory was applied to correct for multiple comparisons at *p* < 0.05 by using the REST software (voxel significance: *p* < 0.001, cluster significance: *p* < 0.05).

The ReHo values of the abnormal brain region were extracted as regions of interest. Furthermore, partial correlation analyses on age, sex, illness duration, time of onset, years of education, and FD as covariates were calculated between abnormal ReHo values and standardized cognitive scores/PANSS scores.

### SVM Analyses

The method of SVM was designed to find the optimal line or surface with the largest interval through an appropriate kernel function to measure the data. This method was widely applied to classify and diagnose the disease (21). SVM was conducted to examine the possibility of abnormal ReHo values in brain regions and to distinguish patients with schizophrenia from healthy controls by using the LIBSVM software package (http://www.csie.ntu.edu.tw/~cjlin/libsvm/) (26). Regarded abnormal ReHo values of brain regions as features, we used the grid search method and Gaussian radial basis function kernels to optimize the parameters, and then the “leave-one-out” cross-validation method was used to calculated the best sensitivity and specificity, finally, permutation test was applied to test the significance of accuracy.

## Results

### Demographic and Clinical Characteristics of the Participants

We recruited 60 inpatients and 52 healthy controls, but only 57 inpatients and 50 healthy controls were enrolled because of the excessive head movement of the three patients with schizophrenia and two healthy controls. The characteristics of the participants are described in Table 1. No difference in age and sex was found between the two groups. The years of education of healthy controls were longer than those of patients with schizophrenia. With regard to the cognitive scores, no difference was found in Wechsler Memory Scale-III Spatial Span, Mayer-Salovey-Caruso Emotional Intelligence Test/Managing Emotions, Working Memory, and Social Cognition between the two groups. However, other cognitive scores were worse in patients with schizophrenia than in healthy controls.

**Table 1:** Demographic and clinical characteristics of the participants.

**Variables**	**Patients with schizophrenia (*n* = 57)**	**Healthy controls (*n* = 50)**	***p***
Age (years)	31.63 ± 11.43	28.38 ± 6.87	0.074
Sex (male/female)	20/37	23/27	0.323
Years of education (years)	12.86 ± 3.42	15.64 ± 2.26	<0.05
Illness duration (years)	2.52 ± 2.72		
TMT-A	36.47 ± 12.71	42.90 ± 9.85	<0.05
BACS-SC	35.23 ± 12.89	45.34 ± 9.29	<0.05
HVLT-R	37.67 ± 13.66	44.52 ± 7.51	<0.05
WMS-III SS	32.51 ± 12.32	34.28 ± 11.22	0.441
NAB Mazes	38.84 ± 10.78	45.44 ± 9.91	<0.05
BVMT-R	42.05 ± 11.61	47.16 ± 9.50	<0.05
Fluency	43.72 ± 11.37	48.76 ± 7.95	<0.05
MSCEIT/Managing Emotions	33.14 ± 8.15	35.32 ± 6.49	0.132
CPT-IP	38.19 ± 13.61	45.56 ± 9.69	<0.05
Speed of processing	34.70 ± 12.12	44.28 ± 9.10	<0.05
Attention/Vigilance	38.19 ± 13.61	45.56 ± 9.69	<0.05
Working Memory	32.51 ± 12.32	34.28 ± 11.22	0.441
Verbal Learning	37.67 ± 13.66	44.52 ± 7.51	<0.05
Visual Learning	42.05 ± 11.61	47.16 ± 9.50	<0.05
Reasoning and Problem Solving	38.84 ± 10.78	45.44 ± 9.91	<0.05
Social Cognition	33.14 ± 8.15	35.32 ± 6.49	0.132
Overall Composite	28.47 ± 13.45	37.60 ± 9.19	<0.05
PANSS Positive	26.39 ± 4.85		
Negative	20.68 ± 6.89		
General	44.79 ± 7.41		
Total	91.84 ± 14.16		

*TMT-A, trail making test part A; BACS-SC, brief assessment of cognition in schizophrenia-symbol coding; HVLT-R, Hopkins verbal learning test-revised; WMS-III SS, Wechsler Memory Scale-III Spatial Span; NAB-Mazes, neuropsychological assessment battery-mazes; BVMT-R, brief visuospatial memory test-revised; MSCEIT, Mayer-Salovey- Caruso Emotional Intelligence Test/Managing Emotions; CPT-IP, continuous performance test-identical pair; PANSS, positive and negative syndrome scale*.

### ReHo Analysis: Differences Between the Patients With Schizophrenia and Healthy Controls

**Figure 1:**
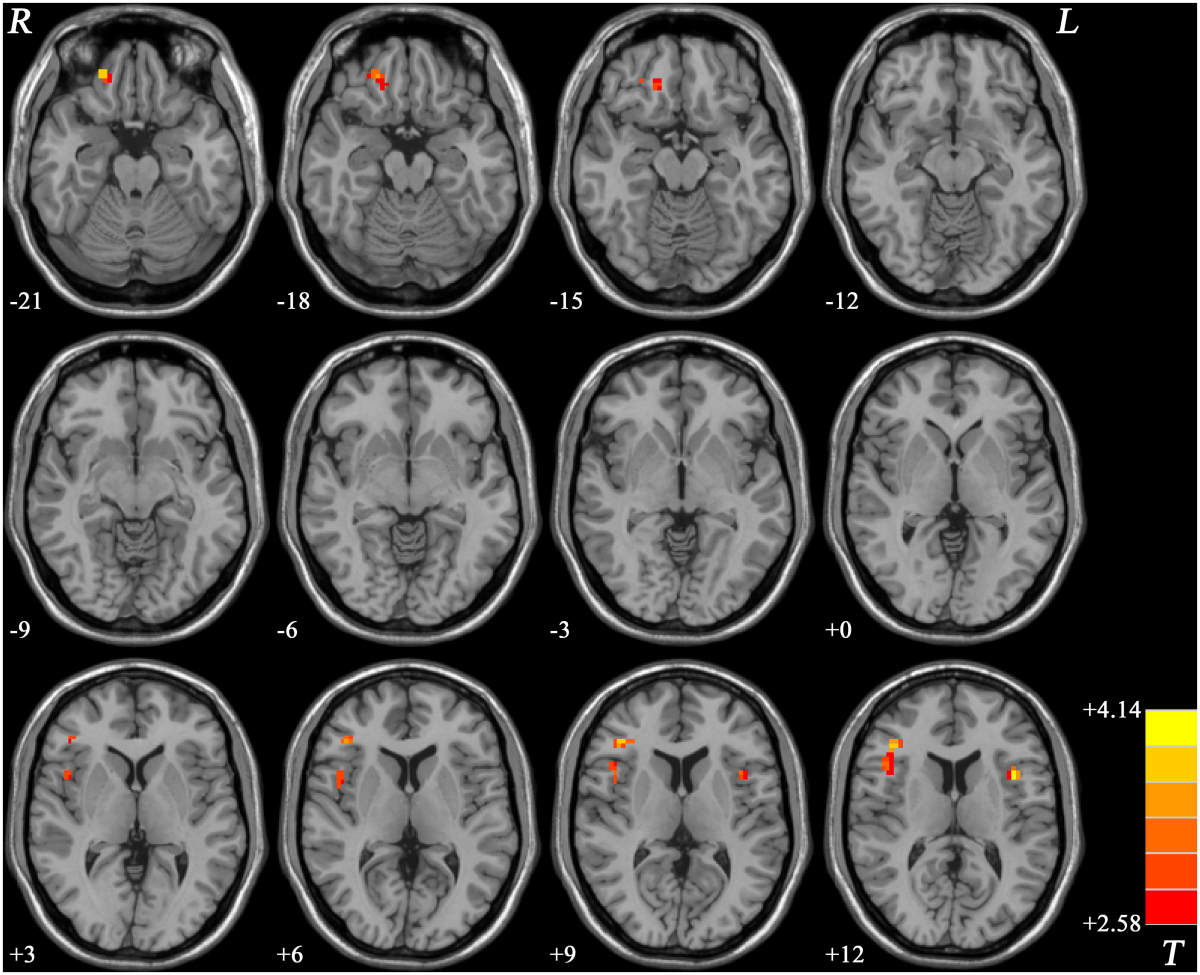
ReHo values difference between patients with schizophrenia and healthy controls. R, right; L, left; ReHo, regional homogeneity.

Patients with schizophrenia showed increased ReHo values in the right gyrus rectus, right inferior frontal gyrus/insula, and left inferior frontal gyrus/insula compared with those of the healthy controls, and no decreased ReHo values in the brain region (Table 2, Figure 1).

**Table 2:** Abnormal ReHo values in the brain region between patients with schizophrenia and healthy controls.

**Cluster location**	**Peak (MNI)**			**Number of voxels**	T value^*^
	**x**	**y**	**z**		
Right Gyrus Rectus	24	45	−21	25	3.8188
Right Inferior Frontal Gyrus/Insula	42	30	12	64	3.8489
Left Inferior Frontal Gyrus/Insula	−39	12	12	23	4.1425

* *A positive t value represents an increased ReHo values*.

*MNI, Montreal Neurological Institute; ReHo, regional homogeneity*.

### Correlation Analysis: Relationship Between the Abnormal ReHo Values and Clinical Characteristics

In patients with schizophrenia, the ReHo values in the right inferior frontal gyrus/insula were significantly and positively correlated with the negative symptom scores (r = 0.319, *p* = 0.023 < 0.05). The ReHo values in the right inferior frontal gyrus/insula were negatively correlated with Hopkins verbal learning test-revised (HVLT-R)/verbal learning (*r* = −0.342, *p* = 0.014 < 0.05). No difference was observed after being corrected by multiple comparisons. On the contrary, no relationship was found between ReHo values and cognition scores in healthy controls (Table 3).

**Table 3:** Relationship between abnormal ReHo values and clinical characteristics.

	**Right inferior frontal gyrus/insula (schizophrenia)**		**Right inferior frontal gyrus/insula (healthy controls)**	
	***p***	**r**	***p***	**r**
HVLT-R	0.014	−0.342	0.277	0.164
Verbal Learning	0.014	−0.342	0.277	0.164
Negative scores	0.023	0.319	/	/

*HVLT-R, Hopkins verbal learning test-revised*.

### SVM Analyses: Identifying Potential Imaging Biomarkers of Schizophrenia

**Figure 2:**
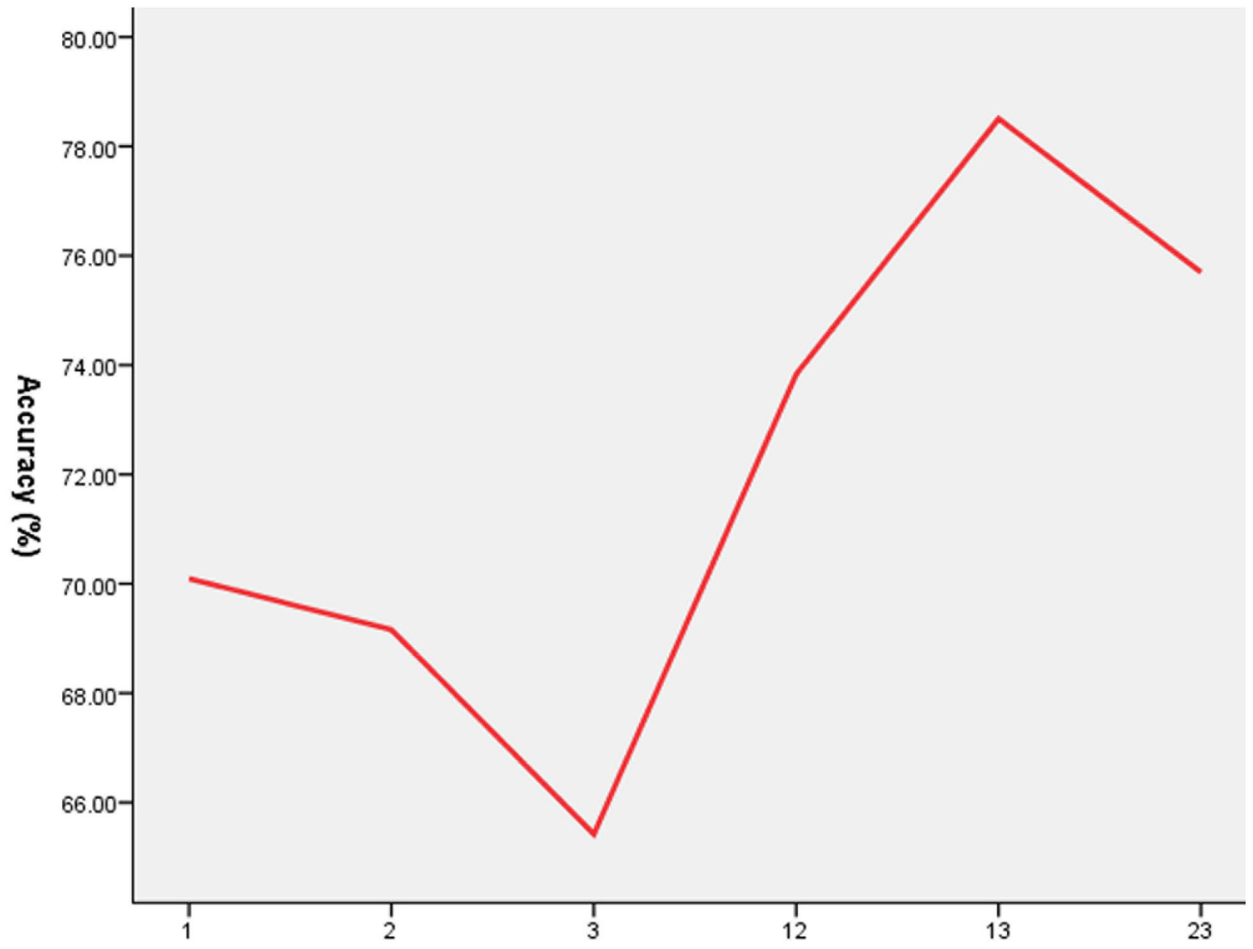
The accuracy of each abnormal ReHo value combination. 1 = Left Inferior Frontal Gyrus/Insula, 2 = Right Inferior Frontal Gyrus/Insula, 3 = Right Gyrus Rectus, ReHo, regional homogeneity.

**Figure 3:**
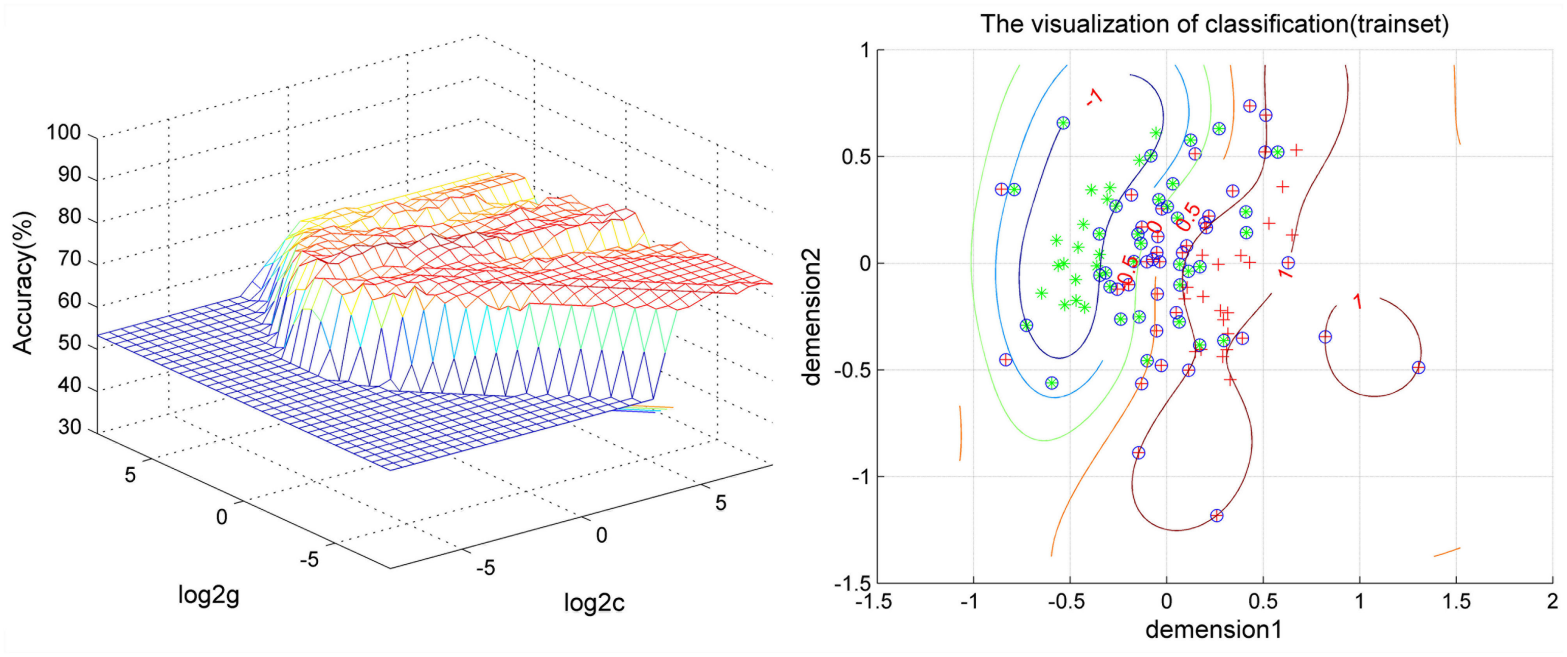
Visualization of the SVM results for distinguishing patients from controls using the combination of ReHo values in the left inferior frontal gyrus/insula and right gyrus rectus. SVM, support vector machine; ReHo, regional homogeneity.

As shown in Figures 2, 3, SVM was used to explore whether abnormal ReHo values could distinguish patients with schizophrenia from healthy controls with high optimal sensitivity and specificity. The accuracy of each abnormal ReHo value was too low to distinguish patients with schizophrenia from healthy controls. Therefore, each abnormality was coupled, and the accuracy of each combination was calculated (Figure 2). Our results indicated that the combination of increased ReHo values in the left inferior frontal gyrus/insula with the right gyrus rectus had 78.5% (84/107) accuracy, 85.96% (49/57) sensitivity, and 70.00% specificity, which were higher than those of other combinations.

## Discussion

As shown in our results, the ReHo values in the right gyrus rectus, right inferior frontal gyrus/insula, and left inferior frontal gyrus/insula increased compared with those of the healthy controls, and all these regions existed in the PFC. However, no decreased ReHo values in the brain regions were found. ReHo could be used to measure the similarity or synchronism of the time series within neighboring voxels and reflect the coordination of regional neural activities. An increased ReHo represented the enhanced neural activity coordination and reflected the abnormal regulation of emotion and behavior. The decreased ReHo indicates uncoordinated movement and disconnection within local neurons in the brain (11). Cognitive dysfunction, including overall composite, speed of processing, attention/vigilance, verbal learning, visual learning, reasoning, and problem solving, was observed in patients with schizophrenia compared with those of the healthy controls. The results indicated that ReHo in the right inferior frontal gyrus/insula was positively correlated with negative symptom scores and negatively correlated with Hopkins verbal learning test-revised/verbal learning, but no difference was found after values were corrected via multiple comparisons. On the contrary, this relationship was not found in the healthy controls.

The prefrontal cortex, which was divided into the dorsolateral PFC (DLPFC), ventrolateral prefrontal cortex (VLPFC), anterior prefrontal cortex, medial prefrontal cortex (MPFC), and orbital PFC (OPFC), plays a crucial role in the cognitive process (particularly in working memory, salience detection, attention, and social cognition) (27) and psychopathology (such as auditory verbal hallucination) (28). A previous review showed an abnormal FC between the PFC and the basal ganglia/temporal regions/parietal regions/hippocampus/default mode network associated with cognition and psychopathology (6). Our results indicated that abnormal ReHo was primarily distributed over the PFC, including the right gyrus rectus (located in OPFC), bilateral inferior frontal gyrus/insula (located in VLPFC), and associated with negative symptom scores in the PANSS and verbal learning ability. Increased ReHo values represented enhanced neural activities coordination in the PFC, supporting that regional abnormalities might affect the remote functional connection and result in cognitive impairment and psychopathology. According to the dopamine hypothesis on schizophrenia, a decrease in dopamine levels in the VLPFC and OPFC is associated with cognitive deficits and negative symptoms (29, 30), which supported our results. Research indicated that ReHo has neurobiological relationship with structural, developmental and neurocognitive (31). In the term of structural MRI, previous studies showed that the gray matter reduction of the PFC is related to prospection impairments in patients with schizophrenia (32). For the functional MRI, increased ReHo values were in the PFC, which is distributed over the DLPFC (19, 28), and MPFC (15, 33), but the parts of the PFC are inconsistent with our results, and those differences may be caused by the type of patients with schizophrenia, the size of sample, the condition of medication, the standard of assessment. In addition to cross-sectional studies, one 8-week follow-up study regarding emotional processing showed that the activation of the VLPFC normalizes after olanzapine treatment (34). Consequently, these findings supported our results that abnormal activities existed in the PFC and possessed the relationship with cognition and psychopathology, further suggesting that abnormal activities in PFC may contribute to the pathophysiology and cognitive impairment in schizophrenia.

To our knowledge, the right gyrus rectus is part of the OPFC and involved in the prefrontal association integration. Todd Lencz et al. (35) emphasized that the rs1344706 polymorphism in ZNF804A (a candidate gene of schizophrenia) may alter neuroanatomical (including the gyrus rectus) and neurocognitive phenotypes; besides, a post-mortem brain mRNA study has revealed that somatostatin mRNA+ cell density of the gyrus rectus layer II is lower in patients with schizophrenia than in healthy controls, which may give rise to the reduced gray matter volume (36). Furthermore, the gray matter volume in the right gyrus rectus decreased in the groups of subjects with an ultrahigh risk of psychosis (37) and first-episode schizophrenia (38, 39). In addition, the gray matter volume of the gyrus rectus are negatively related to the positive scale of PANSS (particularly in delusion scores, suspiciousness/persecution scores, conceptual disorganization scores, grandiosity scores, and hostility scores) in patients with schizophrenia (40). Abnormalities in the right gyrus rectus may occur at the early stage with genetic pre-disposition. Moreover, structural abnormalities affected functional activities in this region. In functional MRI, abnormal FC was found between the cingulate gyrus and gyrus rectus in early-onset schizophrenia (41). Meanwhile, our research indicated that the ReHo in the right gyrus rectus of patients with schizophrenia increased compared with that of the healthy controls; therefore, this damaged region might explain the abnormal FC. Although no association with cognition and psychopathology was found, exploring abnormal neural activities could help us to reveal the mechanism of schizophrenia.

The inferior frontal gyrus/insula possesses core roles in the salience network as well as the cognitive task control network, which is distributed over the VLPFC. In our study, ReHo in the bilateral inferior frontal gyrus/insula increased, but the left frontal gyrus/insula was more severe than the right side. Zhu et al. (42) found that the decreased parameter of asymmetry in the right inferior frontal gyrus/insula, may account for asymmetrical changes in the abnormal ReHo values of this area. Previous studies confirmed that variation in rs1344706 encoding ZNF804A affects structural brain alteration in the inferior frontal (43) and insula (44). Schizophrenia and their unaffected siblings showed the amplitude of low-frequency fluctuation abnormalities in the inferior fronto-insular gyrus and compared it with that of healthy controls (45). Consequently, structural and functional brain abnormalities in inferior frontal gyrus/insula have genetic pre-dispositions, which suggest that functional brain abnormalities in inferior frontal gyrus/insula may be occurred before the onset of disease, and further support neurodevelopmental hypothesis. Moreover, in our study, the ReHo in the right inferior frontal gyrus/insula was positively related to negative symptom scores and negatively associated with HVLT-R scores/verbal learning abilities. However, this relationship was hardly found in healthy controls; therefore, these abnormalities of clinical phenotype might be triggered by increased ReHo values in this region, which might be associated with the enhanced neural activity coordination. This result indicated that ReHo in this area might reflect the severity of negative symptoms and verbal learning abilities. In adolescent-onset patients with schizophrenia, one study showed that the increased FC strength in the right inferior frontal gyrus/insula is related to the general psychopathology scores of PANSS (46). Differences in these results might be caused by various MRI methods and different samples/types/states of patients with schizophrenia (43–45). Furthermore, our results revealed that abnormal neural activities occurred in the inferior frontal gyrus/insula; similarly, Leslie K. Jacobsen et al. (47) found that the glucose metabolic rate in the inferior frontal gyrus/insula of adolescents with childhood-onset schizophrenia increased. Above all the studies implied that abnormalities in the inferior frontal gyrus/insula might be a potential neurophysiological endophenotype of schizophrenia.

In our study, the accuracy of each abnormal ReHo value in the bilateral inferior frontal gyrus/insula or right gyrus rectus was too low to discriminate patients with schizophrenia from healthy controls; this phenomenon may be related to deficits in these regions that may not be specific to schizophrenia (48, 49). Furthermore, coupling each abnormal ReHo value in the regions and using the method of SVM, we found that the combination of increased ReHo values in the left inferior frontal gyrus/insula with the right gyrus rectus had 78.5% (84/107) accuracy, 85.96% (49/57) sensitivity, and 70.00% specificity compared with those of the other combinations. This result might help us distinguish patients with schizophrenia from healthy controls.

A few limitations were found in our study except the sample size and the age range. First, our research was a cross-sectional study. A longitudinal study might help us detect the stability of ReHo and the neural activities of the regions. Therefore, research methods should be optimized, different approaches may be considered to obtain different results, multimodal MRI should be applied to distinguish patients with schizophrenia from healthy controls.

Despite limitations, our study emphasized that hyperactivities were primarily located in the prefrontal regions, including the right gyrus rectus, right inferior frontal gyrus/insula, and left inferior frontal gyrus/insula. Abnormal ReHo values in the right inferior frontal gyrus/insula might reflect the severity of negative symptoms and verbal learning abilities. The combined increases of ReHo values in the left inferior frontal gyrus/insula with the right gyrus rectus might be an underlying biomarker in differentiating patients with schizophrenia from healthy controls.

## Data Availability Statement

All datasets generated for this study are included in the article/supplementary material.

## Ethics Statement

The studies involving human participants were reviewed and approved by the local ethics committee of the Affiliated Nanjing Brain Hospital, Nanjing Medical University (2017-KY017). Written informed consent to participate in this study was provided by the participants' legal guardian/next of kin.

## Author Contributions

NZ and XX designed this research. XX, SG, YM, JW, YG, SN, SL, RZ, and JS collected the imaging data and clinical information. The PANSS scales were evaluated by SG and the MCCB scales were evaluated by SG, YM, JW, and YG. XX and SG analyzed the imaging data. SG wrote the first draft of this manuscript. All authors reviewed and approved the final manuscript.

## Conflict of Interest

The authors declare that the research was conducted in the absence of any commercial or financial relationships that could be construed as a potential conflict of interest.
